# Defect Chemistry, Sodium Diffusion and Doping Behaviour in NaFeO_2_ Polymorphs as Cathode Materials for Na-Ion Batteries: A Computational Study

**DOI:** 10.3390/ma12193243

**Published:** 2019-10-04

**Authors:** Navaratnarajah Kuganathan, Nikolaos Kelaidis, Alexander Chroneos

**Affiliations:** 1 Department of Materials, Imperial College London, London SW7 2AZ, UK; alexander.chroneos@imperial.ac.uk; 2 Faculty of Engineering, Environment and Computing, Coventry University, Priory Street, Coventry CV1 5FB, UK

**Keywords:** NaFeO_2_, defects, Na-ion diffusion, dopants, atomistic simulation

## Abstract

Minor metal-free sodium iron dioxide, NaFeO_2_, is a promising cathode material in sodium-ion batteries. Computational simulations based on the classical potentials were used to study the defects, sodium diffusion paths and cation doping behaviour in the α- and β-NaFeO_2_ polymorphs. The present simulations show good reproduction of both α- and β-NaFeO_2_. The most thermodynamically favourable defect is Na Frenkel, whereas the second most favourable defect is the cation antisite, in which Na and Fe exchange their positions. The migration energies suggest that there is a very small difference in intrinsic Na mobility between the two polymorphs but their migration paths are completely different. A variety of aliovalent and isovalent dopants were examined. Subvalent doping by Co and Zn on the Fe site is calculated to be energetically favourable in α- and β-NaFeO_2_, respectively, suggesting the interstitial Na concentration can be increased by using this defect engineering strategy. Conversely, doping by Ge on Fe in α-NaFeO_2_ and Si (or Ge) on Fe in β-NaFeO_2_ is energetically favourable to introduce a high concentration of Na vacancies that act as vehicles for the vacancy-assisted Na diffusion in NaFeO_2_. Electronic structure calculations by using density functional theory (DFT) reveal that favourable dopants lead to a reduction in the band gap.

## 1. Introduction

There is a demand for high-capacity rechargeable batteries to be used in large scale energy storage devices such as electric vehicles and grid-scale energy storage systems. Lithium ion batteries were of intense interest to achieve this and significant effort has been devoted to explore novel materials to produce high capacity Li-ion batteries [1,2,3,4,5]. However, there is a significant challenge to manufacture Li-ion batteries at large scale because of the low abundance and inhomogeneous distribution of lithium in the world. Furthermore, many lithium rich ores are found in remote areas leaving extraction and transportation difficult. After the commercial success of Li-ion batteries in portable applications, a considerable effort is currently devoted to Li-based supercapacitors, as they exhibit higher energy and power density compared to that of Li-ion batteries and can be used in large scale applications [6,7,8,9,10]. 

Sodium-ion batteries (NIBs) have recently attracted considerable interest because of the high elemental abundance with broader global distribution and the low cost of sodium [11,12,13]. A variety of sodium-based cathode materials, including NaFePO_4_ [14,15,16], Na_2_FePO_4_F [17,18], Na_3_V_2_(PO_4_)_3_ [19,20], Na_3_V(PO_4_)_2_ [21,22], Na_4_Co_3_(PO_4_)_2_P_2_O_7_ [23] and Na_2_CoSiO_4_ [24], have been synthesized and their electrochemical properties studied. There is a continuous active research on synthesizing novel cathode materials for NIBs to improve their capacity and its applicability in electrical vehicles. 

Layered sodium transition metal dioxides—NaMO_2_ (M = Ti, V, Cr, Mn, Fe, Co and Ni) [25,26,27,28,29,30,31,32]—have been proposed as promising electrode materials for rechargeable NIBs due to their high volumetric and gravimetric densities. In addition, NaMO_2_ materials exhibit more transition metal redox compared to their Li analogue, owing to the larger radius of Na ions and multiple staking sequences [33]. 

NaFeO_2_ is an important cathode material for large-scale NIBs, owing to its low cost and environmentally benign nature [34,35]. There are two main polymorphs available for NaFeO_2_: α-NaFeO_2_ (hexagonal) [36] is a layered structure containing sheets of edge sharing FeO_6_ and NaO_6_ octahedrons. Electrode performance of α-NaFeO_2_ was first reported experimentally by Zhao et al. [37]. The electrochemical study by Yabuuchi et al. [38] showed that a reversible capacity of 80–100 mg^−1^ where the flat voltage of 3.3 V vs. Na metal can be delivered. Furthermore, its cycling performance was shown to be a reversible retention of 75% after 30 cycles [38]. Topotactic reaction studies (Fe^3+^/Fe^4+^ redox without the destruction of crystal) reveal that theoretical capacity of 241.8mAhg^−1^ can be achieved in α-NaFeO_2_ [39]. Another experimental study by Okada et al. [40] shows that operating voltage of more than 3.4 V versus Na metal is possible with the Fe^3+^/Fe^4+^ redox. *β*-NaFeO_2_ (orthorhombic) [41] has not been reported yet as an electrochemically active material for NIBs though there are other studies [42,43,44] on this material. 

Atomistic scale simulation simulations based on the classical interatomic potentials can give useful information to the experimentalist on defect chemistry and the Na-ion transport mechanism, together with the activation energies and favourable cation doping of both polymorphs of NaFeO_2_. In previous work [45,46,47,48,49,50,51,52,53,54,55,56,57,58,59,60,61,62], we applied this methodology to promising cathode materials for lithium and sodium-ion batteries. Here, we examine both hexagonal and orthorhombic polymorphs of NaFeO_2_ and calculate the intrinsic defect formation energies, solution energies for a variety of dopants and possible diffusion pathways for sodium-ion conduction. Further, DFT calculations were performed to examine the electronic properties of doped and undoped α-NaFeO_2_.

## 2. Computational Methods 

Classical pair potential calculations based on the Born model for ionic crystals were performed using the generalized utility lattice program (GULP) code [63]. The interionic interactions consist of long-range attraction (Coulombic) and short-range electron–electron repulsion. We used the well-established Buckingham potentials (refer to supplementary information) to model short-range interactions. The atomic positions and the simulation boxes were optimised using the Broyden–Fletcher–Goldfarb–Shanno (BFGS) algorithm [64]. Lattice relaxation around point defects and migrating ions were modelled using the Mott–Littleton method [65]. Vacancy-assisted Na ion diffusion was calculated considering seven interstitial Na ions between local Na hops. Activation energy reported in this study is the local maximum energy along the diffusion path. The present calculation is based on the full ionic charge model within the dilute limit. Therefore, the defect energies will be overestimated, however, the relative energies, and the trends will be consistent [66,67,68]. 

DFT calculations were applied for the electronic properties of NaFeO_2_ by means of the CASTEP plane wave code [69,70]. The generalized gradient approximation (GGA) was applied with the gradient correction added by Perdew, Burke and Ernzerhof (PBE) to the exchange–correlation energy functional [71]. The kinetic energy cut-off of the plane wave basis functions was set at 500 eV and the k-point grid at 3 × 3 × 3 for the geometry optimization calculations. After energy relaxation of the cell, the lattice constants obtained for α-NaFeO_2_ (a = b = 2.96 Å, c = 15.82 Å) are in good agreement with experiment (refer to Table 1). For the calculations of the density of states (DOS), a denser grid (5 × 5 × 5) was applied with a Gaussian smearing of 0.1 eV, taking into account spin polarization. The addition of the Hubbard model is necessary to derive a band gap closer to the experimental one, as the GGA method is expected to underestimate the band gap of insulators [72,73]. The Hubbard+U model was included to account for the Coulombic (repulsive) interaction of the on-site d electrons. It is well known that the GGA description cannot accurately predict the band gap due to electron delocalization overestimation. Therefore, the Hubbard correction term (+U) used for the 3d electrons of Fe and Co in this study is an established empirical method, to account for the strong on-site Coulomb interactions for the calculation of electronic properties. The U parameter was set at 4 eV for the Fe 3d states and at 3.4 eV for the 3d states of the dopant Co, according to literature [72]. This correction brings the energy band gap value closer to the experimental one. 

## 3. Results and Discussion

### 3.1. NaFeO_2_ Crystal Structures

**Figure 1:**
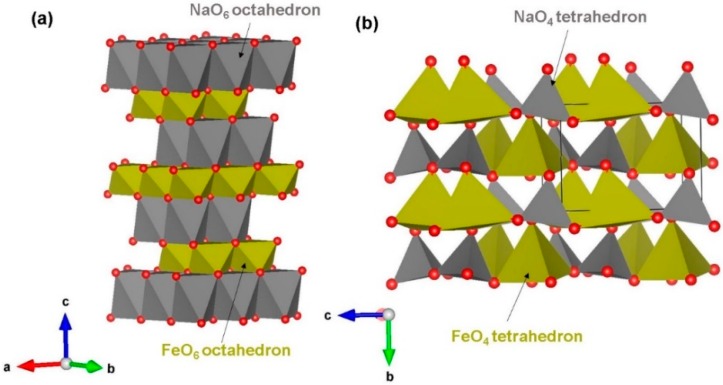
Crystal structures of NaFeO_2_ polymorphs: (**a**) hexagonal (space group ($R\bar{3}$m) and (**b**) orthorhombic (space group Pn2_1_a).

NaFeO_2_ has two different crystallographic structures: *α* (hexagonal, space group R$\bar{3}$m) [32] and *β* (orthorhombic, space group *P*n21a). Hexagonal phase consists of alternate layers of edge-sharing NaO_6_ and FeO_6_ octahedral units along the ab plane, as reported by Takeda et al. [32] (see Figure 1a). The crystal structure of orthorhombic NaFeO_2_ [41] forms corner-sharing tetrahedral units (both NaO_4_ and FeO_4_) in the ac plane as shown in Figure 1b. Using classical pair potentials selected from previous work (refer to Table S1), we first reproduced the experimental structures of both polymorphs. The experimental and calculated structural parameters are listed in Table 1. There is a good agreement between experimental and calculated lattice constants for both structures. Overestimation or underestimation of lattice constants is only within the error margin of ~2% suggesting that defect, diffusion and dopant calculation results would be enough accurate to compare with available experimental data. Furthermore, our calculation suggests that hexagonal phase is 0.31 eV lower in energy than orthorhombic phase.

### 3.2. Intrinsic Defect Processes

Possible defect processes in both α- and β-NaFeO_2_ were calculated. Point defect energies including vacancy and interstitial formation energies were first calculated and then they were combined to calculate the Frenkel- and Schottky-type defect formation energies. These intrinsic defect energies are useful in predicting the electrochemical behaviour of NaFeO_2_. Here we use the Kröger–Vink notation [74] to write equations for the Frenkel, Schottky and antisite defect formation:

(1) $$\mathrm{Na}\;\mathrm{Frenkel}:{\;\mathrm{Na}}_{\mathrm{Na}}^{X}\;\to \;V_{\mathrm{Na}}^{\prime }+{\;\mathrm{Na}}_{i}^{•}$$

(2) $$\mathrm{Fe}\;\mathrm{Frenkel}:{\;\mathrm{Fe}}_{\mathrm{Fe}}^{X}\;\to \;V_{\mathrm{Fe}}^{\prime \prime \prime }+{\;\mathrm{Fe}}_{i}^{•••}$$

(3) $$O\;\mathrm{Frenkel}:{\;O}_{O}^{X}\;\to \;V_{O}^{••}+{\;O}_{i}^{\prime \prime }$$

(4) $$\mathrm{Schottky}:{\;\mathrm{Na}}_{\mathrm{Na}\;}^{X}+{\mathrm{Fe}}_{\mathrm{Fe}}^{X\;}+2{\;O}_{O}^{X}\to \;V_{\mathrm{Na}}^{\prime }+V_{\mathrm{Fe}}^{\prime \prime \prime }+2V_{O}^{••}+{\mathrm{NaFeO}}_{2}$$

(5) $${\mathrm{Na}}_{2}O\;\mathrm{Schottky}:2{\;\mathrm{Na}}_{\mathrm{Na}}^{X}+{\;O}_{O}^{X\;}\to \;2\;V_{\mathrm{Na}}^{\prime }+V_{O}^{••}+{\;\mathrm{Na}}_{2}O$$

(6) $${\mathrm{Fe}}_{2}O_{3}\;\mathrm{Schottky}:\;2{\;\mathrm{Fe}}_{\mathrm{Fe}}^{X}+\;3{\;O}_{O}^{X\;}\to \;2{\;V}_{\mathrm{Fe}}^{\prime \prime \prime }+3{\;V}_{O}^{••}+{\;\mathrm{Fe}}_{2}O_{3}$$

(7) $$\mathrm{Na}/\mathrm{Fe}\;\mathrm{antisite}\;\left(\mathrm{isolated}\right):{\;\mathrm{Na}}_{\mathrm{Na}}^{X}+{\;\mathrm{Fe}}_{\mathrm{Fe}}^{X\;}\to {\mathrm{Na}}_{\mathrm{Fe}}^{\prime \prime }+{\mathrm{Fe}}_{\mathrm{Na}}^{••}$$

(8) $$\mathrm{Na}/\mathrm{Fe}\;\mathrm{antisite}\;\left(\mathrm{clustered}\right):{\;\mathrm{Na}}_{\mathrm{Na}}^{X}+{\;\mathrm{Fe}}_{\mathrm{Fe}}^{X}\to \;\{{\mathrm{Na}}_{\mathrm{Fe}}^{\prime \prime }:{\mathrm{Fe}}_{\mathrm{Na}}^{••}{\text{\}}}^{X}$$

**Figure 2:**
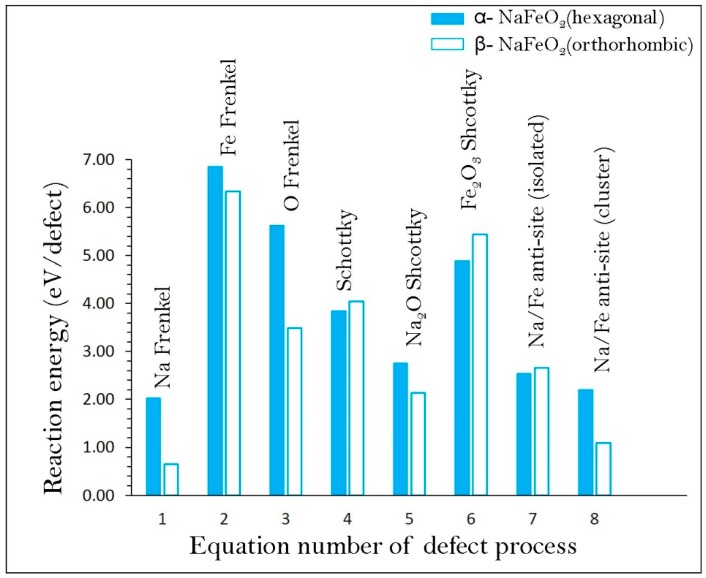
Energetics of intrinsic defect process in α- and β-NaFeO_2_.

Reaction energies for these intrinsic defect processes (refer to Table S2) are reported in Figure 2. The Na Frenkel is calculated to be the most energetically favourable intrinsic defect in both forms of NaFeO_2_. The second lowest defect energy process is found to be the Na–Fe anti-site, suggesting that a small percentage of Na on Fe sites (${\mathrm{Na}}_{\mathrm{Fe}}^{\prime \prime }$) and Fe on Na sites (${\mathrm{Fe}}_{\mathrm{Na}}^{••})$ will be observed at high temperatures. A small distortion is observed in the cation-oxygen bond distances and bond angles in the relaxed structure, but the lattice structure was not altered significantly. There are experimental and theoretical studies showing the presence of anti-site defects in many Li-ion cathode battery materials and in some as-prepared Na ion cathode materials [45,46,48,49,50,51,52,75,76,77,78,79]. There is, however, no experimental report on cation mixing of NaFeO_2_ yet. Nevertheless, in the future experimental preparations of as-prepared structure using different synthetic conditions or during cycling of this material, this defect may be observed. The Frenkel and Schottky defect energies were found to be highly endoergic suggesting that they are unlikely to form at low temperatures. The enthalpy to form Na_2_O Schottky (relation 5) is calculated as 2.76 eV/defect and 2.14 eV/defect for α- and β-NaFeO_2_, respectively. This process can introduce further $\;V_{Na}^{'}$ and $V_{O}^{••}$ in the lattice at elevated temperatures. Conversely, lower defect energetics are observed for β-NaFeO_2_, but the overall trend is retained in both polymorphs. The difference in energetics is mainly due to the different crystal structures and difference in the coordination number of Na and Fe.

### 3.3. Sodium-Ion Diffusion 

In this section intrinsic sodium-ion diffusion of NaFeO_2_ is discussed. Sodium-ion migration with low activation energy is one of the key requirements for a promising high-rate cathode material. The present computational technique allows us to calculate the Na vacancy migration paths together with activation energies, which are difficult to examine by experimental work alone.

**Figure 3:**
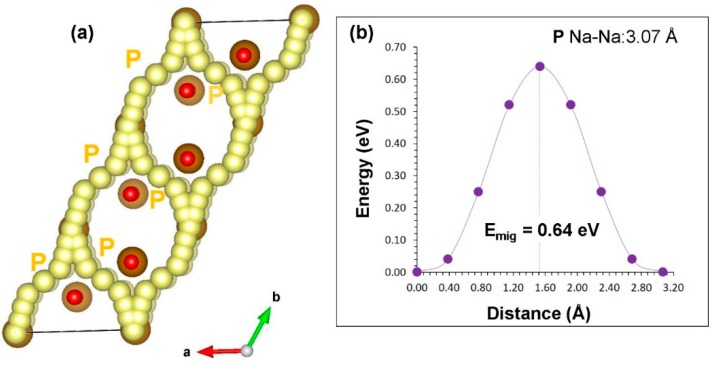
Na ion migration in α-NaFeO_2_: (**a**) long migration paths and (**b**) energy profile of Na vacancy hopping between two adjacent Na sites.

For the Na vacancy migration in α-NaFeO_2_, we identified Na local hops (P) with the jump distance of 3.07 Å, and the migration energy was calculated to be 0.64 eV (refer to Table 2). Long range diffusion paths were then constructed. Sodium-ions migrate in the *ab* plane forming curved paths with overall activation energy of 0.64 eV (refer to Figure 3a). We considered Na hops between the layers but Na–Na migration distances were found to be >5 Å. Figure 3b reports the energy profile diagram for the Na local hop with the activation energy.

**Figure 4:**
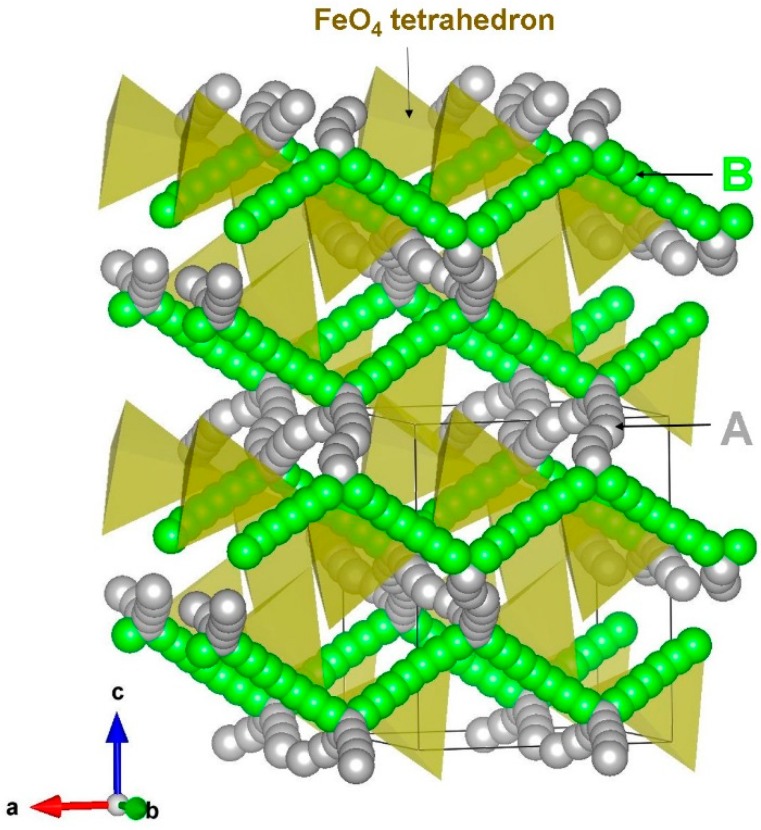
Possible long range sodium vacancy migration paths considered in β-NaFeO_2_. Local Na migration paths are shown in green and grey atoms.

**Figure 5:**
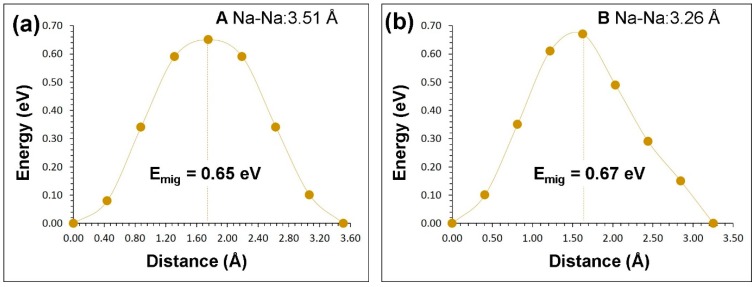
Energy profile diagrams (A and B as shown in Figure 4) for local Na vacancy hopping with Na-Na separation of (**a**) 3.51 Å and (**b**) 3.26 Å β-NaFeO_2_.

Two different local hops, namely, A and B, were identified in β-NaFeO_2_ (refer to Figure 4). The energy profile diagrams for these two hops are shown in Figure 5. The migration path for hop A is in the *bc* plane with the jump distance of 3.51 Å and Na ion moves via a curved trajectory. The activation energy for the hop A is 0.65 eV. In the hop B, Na ions migrate in the *ac* plane with a curved trajectory, but with a jump distance of 3.26 Å and migration energy of 0.67 eV. Three two dimensional long range paths [(A→A→A→A), (B→B→B→B) and (A→B→A→B)] joining local Na hops were identified (see Figure 4). The lowest activation energy (0.65 eV) long range path (A→A→A→A) forms a zig-zag pattern in the *bc* plane. The other two paths have an overall activation energy of 0.67 eV, owing to the presence of local hop B which has an activation energy of 0.67 eV. Here, ions were treated as fully charged. Point defects in a highly ionic material might be expected to be in their fully ionic charge states. The activation energy of migration is defined as the position of the highest potential energy along the migration.

### 3.4. Dopant Substitution

A variety of aliovalent and isovalent dopants were considered on the Fe site. Aliovalent dopant substitutions were charge-compensated by introducing necessary vacancies and interstitials. In all cases, appropriate lattice energies were calculated using the same Buckingham potentials used in this study and used in the solution energy calculations (refer to Table S3). 

First, divalent dopants (M = Ca^2+^, Sr^2+^, Ba^2+^, Mn^2+^, Co^2+^, Ni^2+^, Cu^2+^ and Zn^2+^) were considered. The following reaction equations were used to calculate solution energies by compensating Na interstitials and O vacancies, respectively.

(9) $$2\;\mathrm{MO}\;+2{\;\mathrm{Fe}}_{\mathrm{Fe}\;}^{X}+{\;\mathrm{Na}}_{2}O\;\to 2M_{\mathrm{Fe}\;}^{\prime }+\;2{\;\mathrm{Na}}_{i\;}^{•}\;+\;{\mathrm{Fe}}_{2}O_{3}$$

(10) $$2\;\mathrm{MO}\;+2{\;\mathrm{Fe}}_{\mathrm{Fe}\;}^{X}+{\;O}_{O\;}^{X}\;\to \;2M_{\mathrm{Fe}\;}^{\prime }+\;{\;V}_{O\;}^{••}\;+\;{\mathrm{Fe}}_{2}O_{3}$$

**Figure 6:**
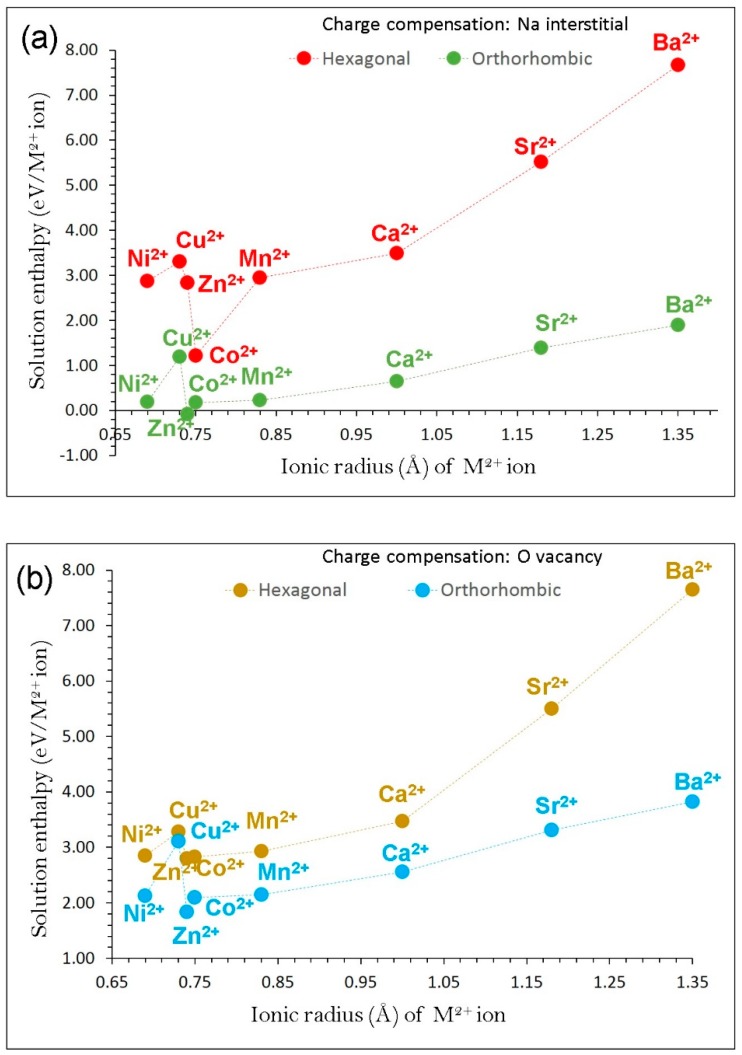
Calculated enthalpy of solution of MO (M = Ni, Cu, Zn, Co, Mn, Ca, Sr and Ba) with respect to the M^2+^ ionic radius in NaFeO_2_ using (**a**) Na interstitial and (**b**) O vacancy as charge compensation.

In the first charge compensation scheme, Na interstitials ions are introduced in the lattice. This can be an efficient way to increase the probability of Na^+^ ion intercalation/de-intercalation processes in the as-prepared NaFeO_2_. Figure 6a reports the solution energies of M^2+^ dopants on the Fe site. Lower solution energies are observed for β-NaFeO_2._ This can be due to the different crystal structures containing different coordination numbers of Fe. The most favourable dopant solution energy (1.22 eV/dopant) is calculated for Co^2+^ in α-NaFeO_2_, suggesting that a possible synthesis–doping strategy to introduce additional sodium into NaFeO_2_ can be achieved by doping Co on Fe sites at elevated temperatures, although the exact amount of Na incorporation cannot be determined. In the case of β-NaFeO_2_, Zn is the energetically favourable dopant with exothermic solution energy (‒0.09 eV/dopant). Other promising dopants are Co^2+^ (0.17 eV/dopant) and Ni^2+^ (0.19 eV/dopant). The possible composition of Co-doped NaFeO_2_ would be Na_1+x_Fe_1−x_Co_x_O_2_ (x = 0.0–1.0). The high solution enthalpy for BaO suggests that Ba^2+^ is an unfavourable dopant to increase Na^+^ ions in both NaFeO_2_ polymorphs. 

In the second charge compensation scheme, the formation of oxygen vacancies is favoured by Zn incorporation in both α- and β-NaFeO_2_ (see Figure 6b). Again, lower solution energies are observed for β-NaFeO_2_ though the values are endoergic. 

Next, we considered trivalent dopants (M = Al^3+^, Co^3+^, Ga^3+^, Mn^3+^, Sc^3+^, In^3+^, Yb^3+^, Y^3+^ and Gd^3+^). Equation 11 was used to calculate the solution enthalpy:

(11) $$M_{2}O_{3}+\;2{\;\mathrm{Fe}}_{\mathrm{Fe}\;}^{X}\to 2{\;M}_{\mathrm{Fe}\;}^{X}+{\mathrm{Fe}}_{2}O_{3}$$

**Figure 7:**
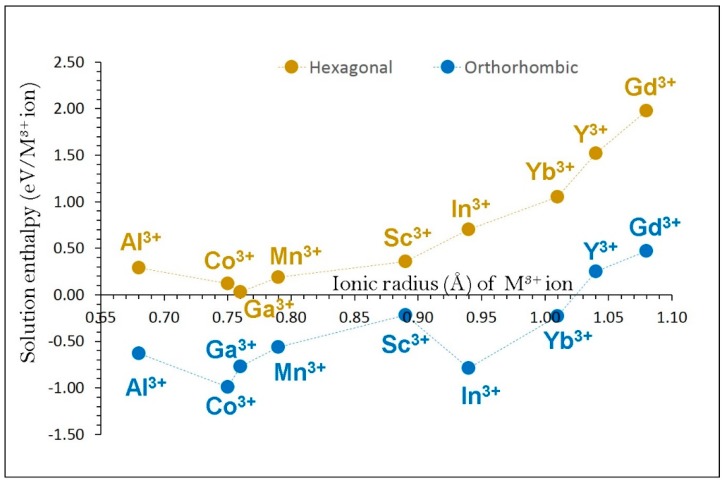
Enthalpy of solution of M_2_O_3_ (M = Al, Co, Ga, Mn, Sc, In, Yb, Y and Gd) with respect to the M^3+^ ionic radius in NaFeO_2_.

Favourable solution energies (0.00–0.20 eV) were noted for Ga, Co and Mn (see Figure 7) in α-NaFeO_2_. Interestingly, exothermic solution energies are observed for all dopants except for Y and Gd in β-NaFeO_2_. The most energetically favourable solution energy (‒0.99 eV/dopant) is observed for Co. 

We considered M^4+^ dopants on the Fe site to increase the concentration of${\;V}_{\mathrm{Na}\;}^{\prime }$in NaFeO_2_. This strategy can facilitate Na self-diffusion via vacancy mechanism. Here, we calculate the solution of MO_2_ via the following equation,

(12) $$2{\;\mathrm{MO}}_{2}+2{\;\mathrm{Fe}}_{\mathrm{Fe}}^{X}+2{\;\mathrm{Na}}_{\mathrm{Na}}^{X}\;\to \;2{\;M}_{\mathrm{Fe}}^{•}+2\;V_{\mathrm{Na}}^{\prime }+{\;\mathrm{Fe}}_{2}O_{3}+\;{\mathrm{Na}}_{2}O$$

**Figure 8:**
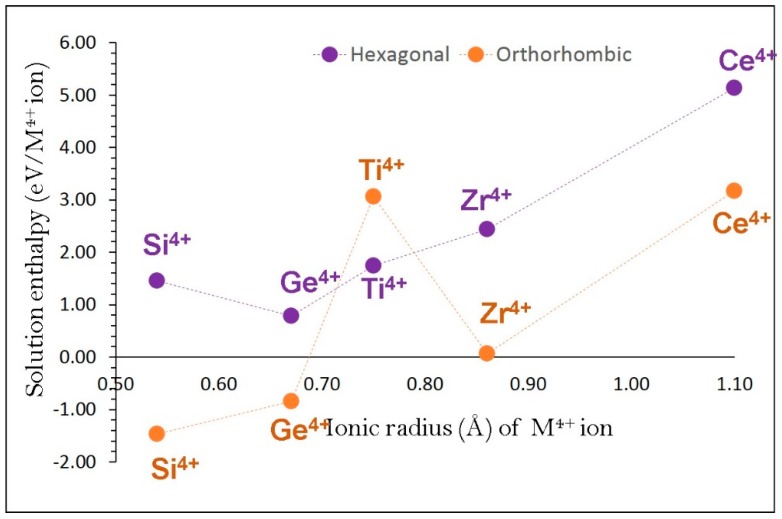
Enthalpy of solution of MO_2_ (M = Si, Ge, Ti, Zr and Ce) with respect to the M^4+^ ionic radius in NaFeO_2_.

Figure 8 reports the solution energies of MO_2_. It is observed that Ge exhibits the lowest solution energy (0.78 eV/dopant) in α-NaFeO_2_. Exothermic solution energies are calculated for SiO_2_ (−1.47 eV/dopant) and GeO_2_ (−0.87 eV/dopant) in β-NaFeO_2_ suggesting that these two dopants should be considered for experimental investigation. 

As redox couples Fe^2+^/Fe^3+^ and Fe^3+^/Fe^4+^ are important for the Na^+^ de-intercalation process and high operating voltage, respectively, a disproportionation reaction was considered according to the following equation,

(13) $$2{\;\mathrm{Fe}}_{\mathrm{Fe}}^{X}\;\to {\;\mathrm{Fe}}_{\mathrm{Fe}}^{•}+{\mathrm{Fe}}_{\mathrm{Fe}}^{\prime }$$

Defect energy for this defect process is −3.47 eV/defect for α-phase and −4.19 eV/defect for β-phase respectively indicating that this process is likely to take place.

### 3.5. Density of States

**Figure 9:**
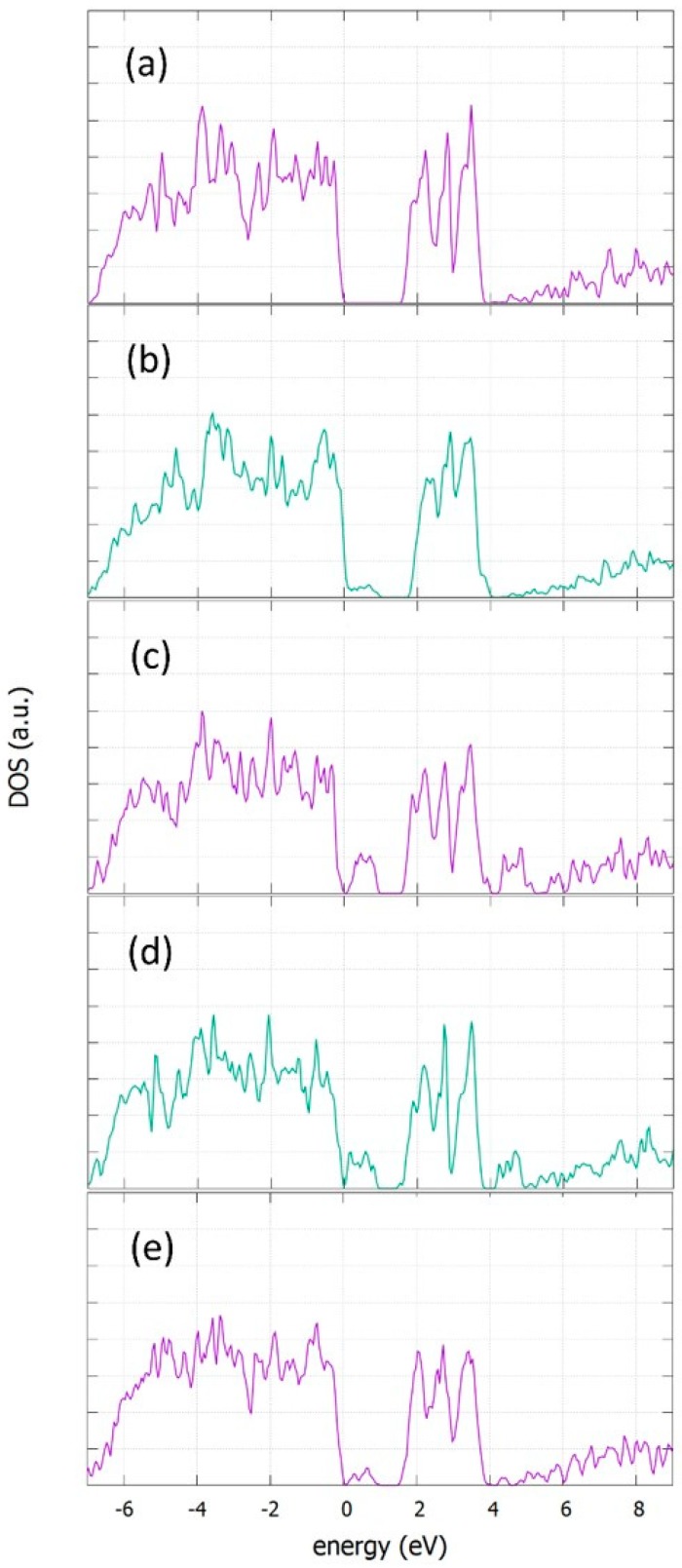
Density of states of (**a**) perfect, (**b**) Co_Fe_- (**c**) Ge_Fe_- (**d**) Si_Fe_- and (**e**) Zn_Fe_-doped α-NaFeO_2_.

The electronic structures of doped and undoped α-NaFeO_2_ were calculated by using first principles calculations, as described in methodology section. A supercell of 2 × 2 × 1 cells was used and the dopants of Co, Ge, Si and Zn were substitutional in the Fe position. This translates to a doping concentration of 8.3%. The density of states (DOS) plots are shown in Figure 9 for α-NaFeO_2_. The incorporation of defects in a substitutional Fe position leads to a band gap reduction and the appearance of defect states near the valence band. The band gap of the perfect structure is calculated to be 1.53 eV. Doping with Co decreases significantly the calculated band gap at 0.8 eV, due to a band tail that is formed near the valence band with a peak at 0.35 eV. Doping with Ge or Si further decreases the band gap at 0.7 eV and 0.6 eV, respectively, due to an appearance of states with peaks at 0.48 eV for Ge and 0.14 eV and 0.56 eV for Si. Doping with Zn also decreases in the same way as the band gap at 0.70 eV, due to states that appear 0.34 eV and 0.60 eV higher that the valence band of the perfect structure. 

## 4. Conclusions

In conclusion, using atomistic simulation techniques, we carried out a systematic survey of both α- and *β*-NaFeO_2_ to investigate intrinsic defects, sodium-ion diffusion paths and favourable aliovalent and isovalent dopants on the Fe site. The present simulations reasonably reproduce the observed polymorphs of NaFeO_2_. The most favourable intrinsic defect type is Na Frenkel. The second most favourable energy defect process is Na–Fe antisite, suggesting that there will be a small population of Na on Fe site and vice versa. The lowest migration energies for long-range Na ion migration in hexagonal (*α*-) and orthorhombic (*β*-) NaFeO_2_ are 0.65 eV and 0.67 eV, respectively, suggesting that both polymorphs exhibit favourable electrode kinetics. The present calculations further suggest that favourable dopants for creating additional Na in the *α*- and *β*-NaFeO_2_ are Co^2+^ and Zn^2+^ on the Fe site, respectively. A high concentration of Na vacancies can be introduced by doping Ge on Fe in *α*-NaFeO_2_ and Si (and Ge) on Fe in *β*-NaFeO_2_ to facilitate the vacancy-assisted Na diffusion in NaFeO_2_. Electronic structure calculations predict that in all cases substitutional doping leads to a reduction in the band gap.

## Figures and Tables

**Table 1:** Experimental and calculated structural parameters for hexagonal (α) and orthorhombic (β) NaFeO_2._

**Hexagonal (** $R\bar{3}$ **m)^32^**			
**Parameter**	**Calc**	**Expt**	**|∆|(%)**
a = b (Å)	3.0687	3.0221	1.54
c (Å)	16.0917	16.0817	0.06
α = β (°)	90.00	90.00	0.00
γ (°)	120.00	120.00	0.00
**Orthorhombic (Pn21a)^41^**			
a (Å)	5.7911	5.6823	1.92
b (Å)	5.3862	5.4258	0.73
c (Å)	7.1186	7.2351	1.61
α = β = γ(°)	90.00	90.00	0.00

**Table 2:** Calculated Na–Na separation and activation energy for the sodium-ion migration between two adjacent Na sites in α-NaFeO_2_ (refer to Figure 3a).

Migration Path	Na–Na Separation (Å)	Activation Energy (eV)
P	3.07	0.64

## Supplementary Materials

The following are available online at https://www.mdpi.com/1996-1944/12/19/3243/s1, Table S1: Interatomic potential parameters used in the atomistic simulations of NaFeO_2_; Table S2: Energetics of intrinsic defect process in NaFeO_2_; Table S3: Solution enthalpy for dopant substation at Fe site in NaFeO_2._
