# Supplementary material for: Defect Chemistry, Sodium Diffusion and Doping Behaviour in NaFeO2 Polymorphs as Cathode Materials for Na-Ion Batteries: A Computational Study
Source: Materials (Basel). 2019 Oct 4;12(19):3243. doi: 10.3390/ma12193243 (PMC6803870; doi:10.3390/ma12193243)
Supplement: Supplementary file 1 [file materials-12-03243-s001.pdf]

materials

MDPI

Supplementary Materials

Defect Chemistry, Sodium Diffusion and Doping Behaviour in $NaFeO_2$ Polymorphs as Cathode Materials for Na-Ion Batteries: A Computational Study

Navaratnarajah Kuganathan [1,2,*] Nikolaos Kelaidis [2] and Alexander Chroneos [1,2]

1 Department of Materials, Imperial College London, London SW7 2AZ, United Kingdom; alexander.chroneos@imperial.ac.uk (A.C.)

2 Faculty of Engineering, Environment and Computing, Coventry University, Priory Street, Coventry CV1 5FB, United Kingdom; ad0636@coventry.ac.uk (N.K.)

* Correspondence: n.kuganathan@imperial.ac.uk

Received: 10 September 2019; Accepted: 2 October 2019; Published: date

Table S1. Interatomic potential parameters used in the atomistic simulations of $NaFeO_2$. Two-body $[\Phi_{ij}(r_{ij}) = A_{ij}\exp(-r_{ij}/\rho_{ij}) - C_{ij}/r_{ij}^6]$.

Interaction	A/eV	ρ/Å	C/eV·Å^6	Y/e	K/eV·Å^{-2}	Lattice Energy (eV)
Na^+–O^{2-}(1)	1497.830598	0.287483	0.000	1.000	99999	–27.59
O^{2-}–O^{2-}(2)	22764.30	0.1490	27.89	-2.860	74.92	---
Fe^{3+}–O^{2-}(3)	1156.36	0.3299	0.00	4.97	304.7	–148.94
Ca^{2+}–O^{2-}(3)	1090.40	0.3372	0.0000	0.7400	34.00	–36.77
Sr^{2+}–O^{2-}(3)	1400.00	0.3500	0.0000	0.6700	21.53	–33.78
Ba^{2+}–O^{2-}(3)	931.79	0.3949	0.0000	0.5400	14.78	–31.33
Mn^{2+}–O^{2-}(3)	715.80	0.3464	0.0000	3.000	81.20	–38.38
Co^{2+}–O^{2-}(3)	696.30	0.3362	0.0000	2.000	10.74	–40.01
Ni^{2+}–O^{2-}(3)	683.50	0.3332	0.0000	2.000	8.77	–40.60
Cu^{2+}–O^{2-}(3)	3799.30	0.2427	0.0000	2.000	99999	–44.70
Zn^{2+}–O^{2-}(3)	499.60	0.3595	0.0000	2.050	10.28	–39.35
Al^{3+}–O^{2-}(3)	1725.20	0.28971	0.0000	3.000	99999	–161.88
Co^{3+}–O^{2-}(3)	1329.82	0.3087	0.0000	2.040	196.30	–157.31
Mn^{3+}–O^{2-}(3)	1267.50	0.3214	0.0000	4.970	304.70	–151.46
Sc^{3+}–O^{2-}(3)	1299.40	0.3312	0.0000	3.000	99999	–145.28
Yb^{3+}–O^{2-}(3)	1309.60	0.3462	0.0000	3.000	99999	–137.45
Ga^{3+}–O^{2-}(3)	1625.72	0.3019	0.0000	3.000	99999	–155.56
In^{3+}–O^{2-}(3)	1495.65	0.3327	4.33	3.000	99999	–141.25
Y^{3+}–O^{2-}(3)	1766.40	0.33849	19.43	3.000	99999	–135.54
Gd^{3+}–O^{2-}(3)	1885.75	0.3399	20.34	3.000	99999	–133.27
Zr^{4+}–O^{2-}(3)	985.869	0.3760	0.0000	1.350	169.617	–109.91
Ce^{4+}–O^{2-}(3)	1986.83	0.3511	20.40	7.700	291.75	–105.31
Si^{4+}–O^{2-}(2)	1283.91	0.32052	10.66	4.000	99999	–128.71
Ge^{4+}–O^{2-}(4)	1497.3996	0.325646	16.00	4.000	99999	–121.57
Ti^{4+}–O^{2-}(5)	5111.7	0.2625	0.00	–0.10	314.0	–124.58

Table S2. Energetics of intrinsic defect process in $NaFeO_2$.

Defect Process/Equation	Reaction Energy/eV	Reaction Energy per Defect/eV	Reaction Energy/eV	Reaction Energy per Defect/eV
	α-NaFeO2		β-NaFeO2	
Na Frenkel /1	4.06	2.03	1.30	0.65
Fe Frenkel /2	13.70	6.85	12.68	6.34
O Frenkel /3	11.26	5.63	6.98	3.49
Schottky /4	15.36	3.84	16.24	4.04
Na_2O Schottky/5	8.28	2.76	6.42	2.14
Fe_2O_3 Schottky/6	24.45	4.89	27.20	5.44
Na/Fe antisite (isolated) /7	5.08	2.54	5.32	2.66
Na/Fe antisite (cluster) /8	4.40	2.20	2.18	1.09

Table S3. Solution enthalpy for dopant substation at Fe site in $NaFeO_2$. The solution energies values calculated using $V_O^{\bullet\bullet}$, as charge compensating defect are given in parentheses.

Dopant	Solution Enthalpy (eV/dopant)	
	Hexagonal	Orthorhombic
Ca^{2+}	3.49 (3.47)	0.64 (2.56)
Sr^{2+}	5.52 (5.50)	1.39 (3.31)
Ba^{2+}	7.67 (7.65)	1.90 (3.82)
Mn^{2+}	2.95 (2.93)	0.22 (2.14)
Co^{2+}	1.22 (2.83)	0.17 (2.09)
Ni^{2+}	2.87 (2.85)	0.19 (2.12)
Cu^{2+}	3.30 (3.28)	1.19 (3.11)
Zn^{2+}	2.83 (2.80)	–0.09 (1.83)
Al^{3+}	0.29	–0.63
Co^{3+}	0.12	–0.99
Ga^{3+}	0.03	–0.77
Mn^{3+}	0.19	–0.56
Sc^{3+}	0.36	–0.22
In^{3+}	0.70	–0.79
Yb^{3+}	1.05	–0.23
Y^{3+}	1.52	0.25
Gd^{3+}	1.98	0.47
Si^{4+}	1.46	–1.47
Ge^{4+}	0.78	–0.84
Ti^{4+}	1.74	3.06
Zr^{4+}	2.44	0.07
Ge^{4+}	5.14	3.17

References

1. Treacher, J.C.; Wood, S.M.; Islam, M.S.; Kendrick, E., Na_2CoSiO_4 as a cathode material for sodium-ion batteries: structure, electrochemistry and diffusion pathways. *Phys. Chem. Chem. Phys.* **2016**, *18*, 32744–32752.
2. Kuganathan, N.; Islam, M.S., Li_2MnSiO_4 Lithium Battery Material: Atomic-Scale Study of Defects, Lithium Mobility, and Trivalent Dopants. *Chem. Mater* **2009**, *21*, 5196–5202.
3. Tealdi, C.; Saiful Islam, M.; Malavasi, L.; Flor, G., Defect and dopant properties of $MgTa_2O_6$. *J. Solid State Chem.* **2004**, *177*, 4359–4367.

4. Kendrick, E.; Islam, M.S.; Slater, P.R., Atomic-scale mechanistic features of oxide ion conduction in apatite-type germanates. *Chem. Commun.* **2008**, *6*, 715–717.
5. Olson, C.L.; Nelson, J.; Islam, M.S., Defect Chemistry, Surface Structures, and Lithium Insertion in Anatase TiO_2. *J. Phys. Chem. B* **2006**, *110*, 9995–10001.

© 2019 by the authors. Submitted for possible open access publication under the terms and conditions of the Creative Commons Attribution (CC BY) license (http://creativecommons.org/licenses/by/4.0/).
